# Polysaccharide-Based Packaging Coatings and Films with Phenolic Compounds in Preservation of Fruits and Vegetables—A Review

**DOI:** 10.3390/foods13233896

**Published:** 2024-12-03

**Authors:** Junkun Pan, Chengheng Li, Jiechao Liu, Zhonggao Jiao, Qiang Zhang, Zhenzhen Lv, Wenbo Yang, Dalei Chen, Hui Liu

**Affiliations:** 1 Zhengzhou Fruit Research Institute, Chinese Academy of Agricultural Sciences, Zhengzhou 450009, China; pjkshihanhan@163.com (J.P.); 19857849561@163.com (C.L.); liujiechao@caas.cn (J.L.); jiaozhonggao@caas.cn (Z.J.); zhangqiang02@caas.cn (Q.Z.); lvzhenzhen@caas.cn (Z.L.); yangwenbo@caas.cn (W.Y.); chendalei@caas.cn (D.C.); 2 Zhongyuan Research Center, Chinese Academy of Agricultural Sciences, Xinxiang 453000, China

**Keywords:** polysaccharide, coating, film, phenolic compound, preservation, fruits and vegetables

## Abstract

Considerable interest has emerged in developing biodegradable food packaging materials derived from polysaccharides. Phenolic compounds serve as natural bioactive substances with a range of functional properties. Various phenolic compounds have been incorporated into polysaccharide-based films and coatings for food packaging, thereby enhancing product shelf life by mitigating quality degradation due to oxidation and microbial growth. This review offers a comprehensive overview of the current state of polysaccharide-based active films and coatings enriched with phenolic compounds for preserving fruits and vegetables. The different approaches for the addition of phenols to polysaccharides-based packaging materials are discussed. The modifications in film properties resulting from incorporating polyphenols are systematically characterized. Then, the application of these composite materials as protectants and intelligent packaging in fruit and vegetables preservation is highlighted. In future, several points, such as the preservative mechanism, safety evaluation, and combination with other techniques along the whole supply chain could be considered to design polyphenol–polysaccharides packaging more in line with actual production needs.

## 1. Introduction

The demand for fruits and vegetables is increasing due to their appealing flavor and high nutritional value, encompassing vitamins, dietary fiber, minerals, and antioxidants [1,2]. The Food and Agriculture Organization of the United Nations (FAO) recommends a daily intake of at least 400 g of fruits and vegetables [3]. However, over one-third of fruits and vegetables are spoiled annually due to microbial infections, environmental factors, and inadequate storage conditions [4,5]. The deterioration in the quality of fruits and vegetables results in various losses related to nutrition, functionality, and sensory characteristics, posing a significant challenge for the preservation industry. Moreover, fruits and vegetables are the primary sources of income and critical drivers of economic development in producing countries. Consequently, there is considerable interest in developing postharvest technologies that aim to extend the shelf life of these products while minimizing spoilage and preserving quality.

Packaging techniques are designed to enhance the shelf life of food products, thereby increasing their availability, affordability, and appeal to consumers [6]. The key characteristics of packaging coatings and films include improved shelf life, guaranteed safety, and superior quality, which can effectively reduce the postharvest losses of fruits and vegetables. However, most packaging currently in use is derived from petroleum-based plastics. Recently, there has been increasing attention paid to the use of eco-friendly alternative materials for packaging, driven by concerns regarding the non-biodegradable nature of petroleum-based products and the diminishing availability of petroleum resources [7,8]. As a result, food scientists and engineers are actively developing novel materials for coatings and films, particularly those derived from abundant renewable sources [9,10]. Biopolymers, such as polysaccharides, proteins, and lipids, present viable alternatives to synthetic packaging materials in preservation applications due to their biodegradability and ability to prevent moisture loss, aroma loss, and oxygen penetration [11]. Polysaccharides, naturally occurring macromolecular compounds with nutritional and antioxidant properties, are particularly well-suited for coatings and films as a substitute for conventional plastic packaging. Additionally, their selective permeability to carbon dioxide and oxygen enables polysaccharide-based edible films and coatings to extend the shelf life of fruits and vegetables [8]. Applying polysaccharide-based coatings and films in food products offers new opportunities for developing innovative and fresh produce packaging systems.

Compared to synthetic plastic-based coatings and films, polysaccharide-based coatings and films have certain limitations, including high hydrophilicity and inadequate mechanical properties. To address these challenges, numerous efforts have been made to enhance the physical performance of polysaccharide-based coatings and films through various strategies, such as modifying the polysaccharides [12], utilizing the layer-by-layer (LBL) assembly technique [13], and incorporating reinforcing fillers [14]. In addition to strengthening mechanical properties and reducing hydrophilicity, using polysaccharides to produce active and smart packaging has emerged as a prominent area of research. One approach involves adding bioactive compounds, such as phenolic compounds or polyphenol-rich extracts, to formulate multifunctional films and coatings [15,16]. Phenolic compounds are widely distributed in various plant sources, such as fruits, vegetables, and cereal grains, and they exhibit considerable functional and structural diversity [17]. Given their unique functional groups, phenolic compounds can be incorporated into polysaccharide films to improve their functional and mechanical properties [18]. Furthermore, the addition of phenolic compounds into polysaccharide-based coatings and films would enhance their antimicrobial activities, as they show a significant ability to control the microbial growth and retard the spoilage of fruits and vegetables.

Researchers have increasingly utilized phenolic compounds as active agents in developing edible eco-friendly packaging materials over the past few decades. To the best of our knowledge, no systematic reviews or subsequent research reports have comprehensively assessed the updated data on phenolic compounds as additives in polysaccharide-based coatings and films for preserving fruits and vegetables. This review aims to analyze the latest trends and prospects for using edible polysaccharide-based films and coatings with phenolic compounds to enhance the safety, quality, and nutritional properties of fresh fruits and vegetables. This review begins with a systematic summary of the preservation properties of polysaccharides and phenolics that have been used in fruits and vegetables postharvest storage, respectively. Additionally, the incorporation approaches and the improvement of packaging properties by adding phenolic compounds are discussed. Finally, the recent applications of these composite packaging materials in preserving fruits and vegetables are summarized, and their development trend is highlighted.

## 2. Characteristics of Selected Polysaccharides

**Figure 1:**
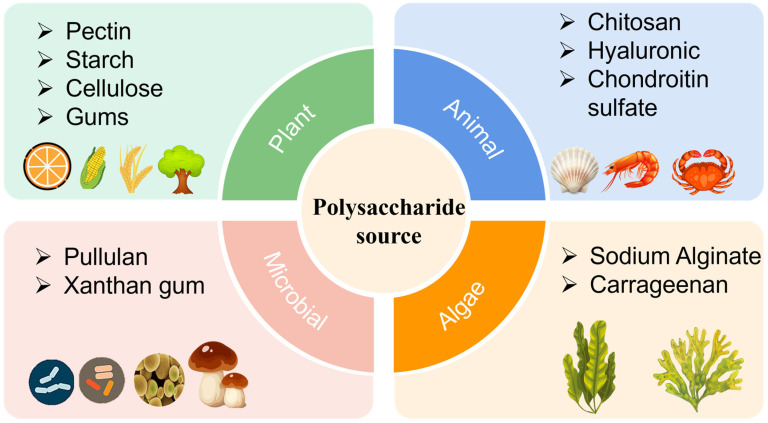
Classification of polysaccharides according to their sources.

Polysaccharides are natural polymers composed of the same or different types of monosaccharides, linked in linear form or branched chains. As shown in Figure 1, typical polysaccharides can be classified based on their source into plant polysaccharides (such as pectin, starch, cellulose, and gums), animal polysaccharides (including chitosan, hyaluronic acid, and chondroitin sulfate), microbial polysaccharides (pullulan and xanthan gum), and algae polysaccharides (such as sodium alginate and carrageenan) [19,20]. These polysaccharides differ in structure and functionality but often exhibit strong film-forming capabilities. They have been developed into films and coatings for packaging various foods, including meats, fish, fruits, and vegetables.

### 2.1. Plant Polysaccharides

#### 2.1.1. Pectin

Pectin is a complex polysaccharide primarily found in plants, characterized by its high molecular weight and branched structure composed of *β*-(1,4)-D-galacturonic acid. The molecular conformation of pectin, which is crucial for its physical properties, consists of three main regions: homogalacturonan (HGA), rhamnogalacturonan I (RG-I), and rhamnogalacturonan II (RG-II) [21]. The ratio of these regions in pectin determines its physical properties, including its gelling capabilities. Pectin can be categorized into high-methoxyl (HM) pectin and low-methoxyl (LM) pectin based on the degree of esterification. HM pectin forms gels in the presence of high soluble solids and a low pH, while LM pectin gels in the presence of divalent cations such as calcium [22]. These gels are highly stable and possess water-resistant properties, which are beneficial for food preservation. The viscosity of pectin solutions is also an important factor; pectin with a higher content of linear structure tends to have higher viscosity, which is advantageous for film or coating formation. Pectin-based packaging offers significant advantages in extending the shelf life of food by delaying lipid oxidation, controlling water movement, and inhibiting microbial growth [23]. Edible coatings and films made from pectin have been successfully employed in prolonging the shelf life of various fruits and vegetables [24]. Additionally, researchers have explored pectin-based coatings enriched with functional components such as polyphenols, essential oils, antagonistic microorganisms, plant extracts, and nanoparticles to enhance the antimicrobial properties and efficiency of the pectin films. For instance, Eça et al. [25] extracted aqueous and alcoholic extracts from five distinct fruits (*Malpighia glabra*, cashew apple, papaya, pequi, and strawberry) and incorporated these extracts into pectin films, thereby enhancing the antioxidant capacity while preserving the physical properties of the films. Quiroga et al. [26] incorporated an antagonistic yeast (*Cryptococcus laurentii*) into a pectin coating to enhance the postharvest protection of apples against *Penicillium expansum*. Li et al. [27] fabricated a pectin/gelatin film loaded with curcumin and silver nanoparticles (AgNPs) capable of monitoring the freshness of packaged shrimp based on the variation of pH values during spoilage. Rodriguez-Garcia et al. [28] added oregano essential oil into pectin coatings to prevent *Alternaria alternata* decay and improve the antioxidant capacity of postharvest tomatoes. These advancements highlight the potential of pectin in creating sustainable and effective food packaging solutions.

#### 2.1.2. Starch

Starch, the reserve polysaccharide of most plants, can be extracted from cereals (corn, wheat, or rice) or tubers (potato or cassava) as a hydrocolloid polymer. The polymer’s backbone consists of amylose and amylopectin, which provide starch granules with varied sizes, shapes, and functionalities. Amylose-rich starches, characterized by their linear *α*-1,4 glycosidic bond structure, are preferred for edible coatings and films, while amylopectin features a branched structure with α-1,6 glycosidic bonds, adding complexity to its framework. It excels in food preservation due to its biodegradability, high biocompatibility, and relatively low cost [29]. However, the low tensile strength, poor water resistance, and no-antibiosis properties limit the application of starch-base films in food preservation [30]. Blending starch with other polymers with very good mechanical, physical, and bioactive properties can overcome this dilemma. Mixing with hydrophobic polymers, such as poly-vinyl alcohol (PVA), could improve the water-resistance properties significantly [31]. By introducing antimicrobial substances into the starch matrix, it can be used as an active packaging material to reduce, inhibit, or delay the growth and reproduction of microorganisms that may exist on the surface of food and extend the shelf life. Deng et al. [32] successfully synthesized quaternary ammonium salt modified amylose starch/chitosan antibacterial films. The results showed that the proton amine groups on the film were closely adsorbed to the bacterial surface through electrostatic interactions, inhibiting the normal physiological functions of the cell membrane and effectively suppressing bacterial growth and reproduction. Starch films also exhibit enhanced mechanical and tensile properties when natural additives are incorporated. Li et al. [33] evaluated the properties of antimicrobial starch-based coatings infused with essential oils and found that this edible coating system enhanced the extensibility and improved its barrier properties. At present, many studies use inorganic nano components to achieve surface micro nano level roughness construction, while forming a hierarchical aggregation state during the process, thereby achieving hydrophobic or superhydrophobic effects on the film surface [34].

#### 2.1.3. Cellulose and Its Derivatives

Cellulose is the most abundant renewable polymer on the earth, primarily sourced from plant-based materials such as cotton, wood, flax, and straw. Comprising glucose monomers linked by β-1,4 bonds, cellulose is notable for its ability to withstand substantial mechanical stress and high temperatures [35]. However, pure cellulose is not soluble in water, and the corresponding films are prepared by dissolving cellulose in ionic liquids or under harsh conditions (e.g., prolonged immersion in NaOH/urea/water), followed by stirring at low temperatures to obtain a cellulose solution. Thus, cellulose derivatives have been used as alternatives for manufacturing corresponding films [36]. These derivatives are mainly classified into cellulose ether and cellulose ester derivatives. Cellulose ether derivatives, such as methyl cellulose (MC), hydroxypropyl methylcellulose (HPMC), and carboxymethyl cellulose (CMC), can be used for coating fruits. CMC is the most commonly used water-soluble cellulose ether derivative and it is widely applied in the food industry [37]. Ali et al. [38] studied the effect of carboxymethylcellulose CMC (1%) coating on the quality of ‘Kinnow’ mandarins under refrigeration at 5 ± 1 °C for 30 days. Compared to the control group, the CMC treatment inhibited disease incidence and significantly increased the activity of antioxidant enzymes, thereby ensuring fruit quality. In contrast, cellulose ester derivatives, such as cellulose acetate and cellulose phthalate, are usually used to make films, which are less likely to form gels in water, and which are therefore widely used in semi-permeable microporous films [39]. Liang et al. [40] successfully developed a polylactic acid-cellulose acetate-phenyl salicylate-based food packaging film by using cellulose acetate as a plasticizer and phenyl salicylate as an antimicrobial agent, and found that this packaging film could retard the decay of lilies and extend their shelf life. In addition, due to the hierarchical organization of cellulose, cellulose nanoparticles can be efficiently separated from cellulose by mechanical and chemical methods or a combination of both [41]. Cellulose nanocrystals (CNCs) can also be used to prepare edible coatings or films. For instance, incorporating CNCs into a chitosan (CS) film applied to mangoes resulted in a 202% increase in barrier properties against oxygen and a 63% increase against water vapor [42].

#### 2.1.4. Plant-Based Gums

Gums are high molecular weight polysaccharides composed of glucose, fructose, mannose, and other sugars, and they are characterized by their sustainability, biodegradability, and biosafety [43,44]. Plants are the primary source of gums, which are derived from plant cell walls, tree secretions, seed endosperm, and tubers. Plant-based gums are mainly classified into two main groups: exudate gums (e.g., arabic gum, karaya gum, and ghatti gum) and seed gums (e.g., guar gum and acacia bean gum). Due to their texturizing properties, gums are widely used in forming edible coatings. These coatings are typically applied to the surface of fruits and vegetables through dipping or spraying, followed by air drying [45]. Gum-based coatings inhibit the activity of softening enzymes in fruits and vegetables, reducing postharvest oxidative stress and postharvest quality loss. For example, during persimmon storage, the gum arabic coating inhibited the activity of softening enzymes, such as polygalacturonase (PG) and pectin methyl esterase (PME), maintained the activity of antioxidant enzymes, delayed the softening of fruits and vegetables, and prolonged storage time [46]. Interestingly, combining two or more gums can yield synergistic benefits. Mostafavi et al. [47] demonstrated that binary solutions of gum tragacanth and locust bean gum, prepared in various mixing ratios, exhibited significant synergism in viscosity and lower surface tension compared to pure gum solutions, making them ideal for use as edible coatings for fruits, vegetables, and other food products.

### 2.2. Animal Polysaccharides

#### 2.2.1. Chitosan

Chitosan (CS) is a natural polymer derived from chitin, which is present in the exoskeleton of crustaceans such as crabs and shrimp [48]. This linear polysaccharide is composed of β-(1-4)-2-acetamido-D-glucose and β-(1-4)-2-amino-D-glucose units. It exhibits effective film-forming properties related to its molecular structure and chemical characteristics. CS molecules are rich in hydroxyl and amino groups, which can form hydrogen bonds with water molecules, allowing CS to create colloidal solutions in weakly acidic environments. As the water in these solutions gradually evaporates, hydrogen bonding interactions between CS molecules are strengthened, resulting in films with specific strength and elasticity. Chitosan is of interest as a potential food preservative owing to its antimicrobial activity against broad bacteria and fungi [49]. Due to the interaction between active hydroxyl and amino groups, chitosan possesses a strong antioxidant capacity by scavenging hydroxyl radicals. Additionally, CS is involved in the immune signaling network of fruit and regulates disease resistance pathways through hormones [50]. Antioxidant enzymes, cell wall degradation enzymes, and sugar metabolism enzymes could be remarkably influenced by CS, as it reduces the oxidative stress to prevent browning, delaying softening and alleviating chilling injury [51,52,53]. The use of CS coatings delayed the spoilage of strawberries, maintained high levels of superoxide dismutase (SOD) and catalase (CAT) activity, and regulated genes related to sucrose metabolism, titratable acid accumulation, disease resistance, as well as the synthetic metabolism of jasmonic acid and abscisic acid pathways [50]. Lin et al. [52] found CS-treated longan exhibited lower fruit respiration rates, reduced peel browning, and decreased disease indices compared to the control, with optimal results observed at a dilution of 1:500. Similarly, Xin et al. [54] reported that a 2% CS coating significantly prolonged the softening time of sweet cherries by 6.4% and maintained sodium carbonate-soluble pectin (SSP) content, which was 6.6% higher than the control. These findings suggest that CS coating is a simple and effective postharvest treatment for improving fruit storage quality. On the other hand, CS can be easily combined with other components to form films, such as other polysaccharides, plasticizers, proteins, and lipids, and this can increase the properties of the composite film. Sabaghi et al. [55] demonstrated that increasing the CS content in Kefiran/chitosan composite films resulted in a tighter structure, reduced water vapor permeability, and increased antioxidant activity. The chitosan/thyme oil coating combined with UV-C treatment inhibited the gene expression of PG, PME, and cellulase, reduced their activities and then delayed the ripening and senescence of blueberries [56].

#### 2.2.2. Hyaluronic Acid

Hyaluronic acid (HA), an acidic mucopolysaccharide, consists of repeating disaccharide units composed of D-glucuronic acid and *N-*acetylglucosamine, linked by β-(1-3) glycosidic bonds. The abundance of hydroxyl groups in its structure allows HA, across a range of molecular weights, to form hydrogels by hydrogen bonding with water molecules, thus ensuring the stability of the polymer system in preservation materials. The physicochemical and biological properties of HA can be tailored through chemical modifications and conjugations with other molecules. Sturabotti et al. [57] demonstrated that conjugating thymol with HA produced a compound with potent antifungal activity. Additionally, integrating HA with curcumin enhances the stability, bioavailability, and antioxidant activity of otherwise insoluble antioxidants while also exerting inhibitory effects on the growth of *Salmonella*, *Bacillus subtilis*, and *Staphylococcus aureus* [58]. This combination shows promise as a novel film-aiding agent, enhancing the bacteriostatic properties of films. Al-Hilifi et al. [59] introduced HA in fruit preservation coatings for the first time, developing an edible polysaccharide-protein coating based on HA for strawberry preservation. The results indicated that the inclusion of HA significantly enhanced the antioxidant properties of the coating in a dose-dependent manner, positively impacting the intrinsic quality of the coated strawberries. These applications underscore the potential of HA to extend the shelf life and maintain the nutritional integrity of fresh produce.

#### 2.2.3. Chondroitin Sulfate

Chondroitin sulfate consists of *N*-acetylgalactosamine and glucuronic acid units and it is prominently found in animal cartilage [60]. The presence of numerous sulfate groups imparts a hydrophilic character to its surface, providing robust colloidal stability during complex formation. This attribute has been harnessed to enhance the stability and activity of anthocyanins within chondroitin sulfate-based nanocomposites. The capacity of chondroitin sulfate to augment and stabilize anthocyanin color in composite film systems has been previously reported, showcasing its utility in developing novel pH-sensitive and colorimetric indicator films for monitoring shrimp freshness [61]. Furthermore, research has established chondroitin sulfate as an effective carrier for encapsulating phenolic compounds, facilitating structural homeostasis and controlled release, particularly beneficial for film applications [62,63]. In recent advancements, Yu et al. [64] encapsulated anthocyanins within chondroitin sulfate-chitosan nanoparticles and integrated these into chitosan films, effectively inhibiting the growth of *Escherichia coli* and *Staphylococcus aureus*. These findings highlight the potential of chondroitin sulfate in active food packaging, contributing to the development of materials with enhanced functionality and extended shelf life.

### 2.3. Microbial Polysaccharides

#### 2.3.1. Pullulan

Pullulan is predominantly obtained through the fermentation of certain fungi and yeast. Its structure is composed of maltotriose units linked by *α*-1,6 glycosidic bonds, and it is highly soluble in both hot and cold water. Due to its non-toxic, biodegradable, water-soluble, odorless, tasteless, and edible properties, pullulan is considered a suitable polysaccharide for film preparation in the food industry. The coating forms a transparent and flexible layer that effectively resists moisture and gases that degrade fruits. Strawberries packaged with pullulan ester films exhibited a significant reduction in weight loss, maintained firmness, and demonstrated a longer shelf life [65]. Chang et al. [66] developed antimicrobial pullulan fiber (APF)-based packaging using focused rotary jet spinning that is biodegradable and capable of wrapping food substrates. APF-coated avocados showed less weight loss and extended shelf life as the proliferation of natural microflora was inhibited. Combining pullulan with other substances, new mechanical and functional properties can be achieved to meet the usage requirements. The elongation at break of oat protein/pullulan composite film increased by 52.49% compared to the pure pullulan film and the addition of nisin remarkably increased its antimicrobial properties [67]. Films made from carboxymethyl chitosan (CMCS) and pullulan incorporating galangal essential oil (GEO) effectively preserved mango fruits during 15 days of storage at 25 ± 1 °C [68].

#### 2.3.2. Xanthan Gum

Xanthan gum (XG) is an exopolysaccharide produced by Xanthomonas campestris cultures under aerobic conditions. XG has a high viscosity and readily disperses in water [69]. Numerous studies have investigated its application in edible films and coatings. It can resist ultraviolet light and can improve the mechanical and thermal stability of highly transparent films [70,71]. Its exceptional resistance to enzyme degradation renders it an effective coating for both fresh-cut and whole fruits, providing long-lasting protection. Therefore, the addition of XG into food products can fulfill the dual purpose of extending shelf life and enhancing gloss. Previous studies have investigated the application of XG coatings when storing various fresh fruits, including strawberries, tomatoes, pears, and bananas [72]. Due to the intermolecular interactions and viscosity synergy between XG and other biomaterials, it is often mixed with other polymers to improve the water resistance in binary or ternary composite materials, such as CMC and CS [70,73]. Lara et al. [74] studied spraying coatings of XG/citric acid/glycerol composite solution on fresh-cut lotus roots, and found it effectively reduced browning and inhibited the growth of *Bacillus subtilis*.

### 2.4. Algae Polysaccharides

#### 2.4.1. Sodium Alginate

Sodium alginate (SA) is a natural polysaccharide derived from brown seaweed. It is widely used for various biomedical applications due to its colloidal properties, which are characterized by the composition of mannuronic acid and guluronic acid units [75]. The disadvantages of this biopolymer are its low stability in humid environments and its high sensitivity to degradation. SA has been modified in various ways, such as adding metal ions (Cd(II), Cu(II), and Pb(II)) [76] or combining it with polymers such as cellulose [77] or chitosan [78]. Wang et al. [79] reported that the composite films prepared by blending SA with whey protein isolate had a relatively dense structure, thereby reducing the water vapor permeability (WVP). Hydrogel films (HGF) produced by methyl chitosan, SA, and citrus pectin through the electrostatic interaction of opposite charges between polysaccharides exhibited excellent low moisture permeability. These HGF significantly delayed strawberry ripening, dehydration, microbial invasion, and respiration [80]. The combination of SA with other polysaccharides as composite coatings acted out preferable preserving benefits. Adding antimicrobial compounds is also a novel method for improving the preservation properties of coatings and films derived from SA. The incorporation of sage (*Salvia sclarea*) essential oil in SA films caused an increasing antioxidant activity of the films [81]. Similar results were found when tannic acid (TA) was added to SA films. The bioactive films exhibited better WVP, remarkable UV-light barrier ability, and elevated antioxidant and antimicrobial activity against *Escherichia coli* [82].

#### 2.4.2. Carrageenan

Carrageenan is a natural polymer synthesized from red algae (*Eucheuma spinosum* and *Eucheuma cottonii*) using separation or hydrolysis. Its structure consists of disaccharide units linked by α-1,3 and β-1,4 bonds from 3,6-anhydro-D-galactose and D-galactose [83]. Due to its biodegradability and ease of extraction, carrageenan is utilized for synthesizing films and coatings in the preservation industry. The properties of carrageenan can be enhanced by adding components when used as an edible coating. The cyclodextrins and maltodextrin have always been used to affect the tensile strength and elongation of carrageenan/konjac glucomannan mixed gel [84,85]. Studies have shown that carrageenan-zinc oxide nanocomposite coatings can reduce physical and biological damage to mangoes [86]. On the 19th day, the control mangoes showed skin wrinkles, while the coating fruits were still able to maintain their firmness. Carrageenan coatings and films can similarly benefit vegetable storage, as tomatoes coated with starch and carrageenan films have performed better than unwrapped tomatoes [87].

## 3. Mechanism of Phenolic Compounds in Food Preservation

**Figure 2:**
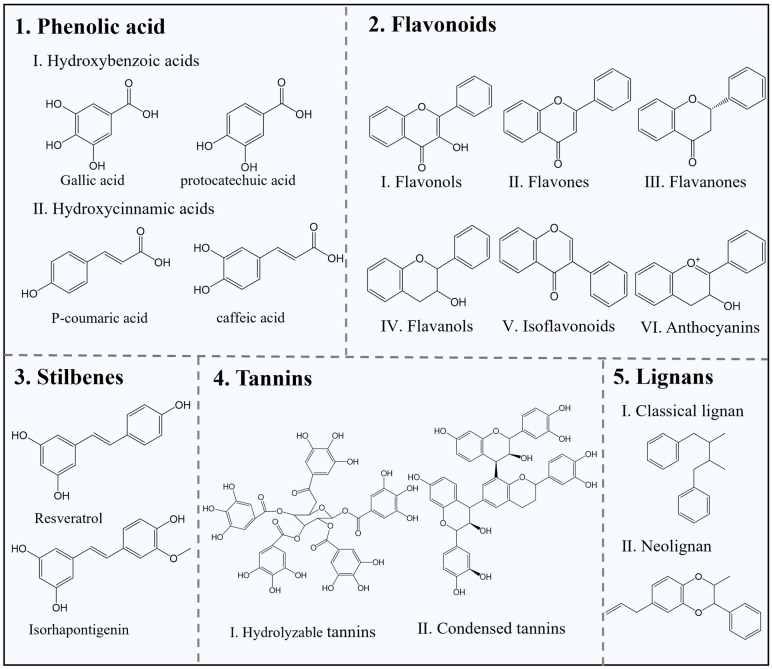
Classification of phenolic compounds.

Phenolic compounds are a class of phytochemicals that are secondary metabolites in plants, naturally occurring in vegetables, fruits, and grains [88]. These compounds exhibit various structures, yet they share a common characteristic of containing at least one aromatic ring and one or more hydroxyl groups. Phenolic compounds can be classified based on the number of phenolic rings, including phenolic acids, flavonoids, stilbenes, tannins, and lignans [89] (Figure 2). Typically, phenolic compounds possess antioxidant and antibacterial properties, and their unique molecular structure makes them important components in active packaging and edible films/coatings [90]. They not only help maintain the physicochemical properties of food and enhance its sensory attributes but also prevent oxidation and extend shelf life by inhibiting microbial growth. The following subsections will discuss two primary properties of phenolic compounds in preserving fruits and vegetables: antioxidant and antimicrobial properties (including antibacterial and antifungal effects), thereby illustrating the significance of phenolic compounds in edible films and coatings.

### 3.1. Antioxidant Properties

The oxidation and browning of nutrients induced by the oxidation reactions produces some secondary and potentially toxic compounds affecting nutritional quality and food safety. Active substances with antioxidant activities could inhibit or delay food oxidation by limiting the occurrence or spread of oxidative reactions. Phenolic compounds have been widely considered to be very good natural antioxidants in food preservation, exhibiting different activity levels due to their varying chemical structures. The antioxidant mechanism is attributed to the presence of multiple hydroxyl groups in their molecular structure, enabling them to trap free radicals effectively, chelate metal ions, inhibit oxidative reactions, and reduce oxidative damage [91]. In addition, phenolic compounds can also regulate the activity of oxidation-related enzymes by influencing signal transduction pathways. Therefore, phenolic compounds can slow down browning of fruit and vegetables and extend their shelf life. Litchi is particularly susceptible to browning due to the accumulation of reactive oxygen species (ROS) and increased polyphenol oxidase activity in its pericarp. Tea polyphenols can inhibit or delay browning during the storage of litchi by suppressing polyphenol oxidase enzyme and reducing lipid peroxidation, which is a key factor contributing to rancid odor [92]. Similarly, the browning process in mashed potatoes and apples, which is also attributed to polyphenol oxidase activity, can be inhibited by the phenolic components of rice bran extract, with *p*-coumaric acid being identified as the active antioxidant [93]. Basanta et al. [94] extracted phenolic compounds from cherries and applied them to develop a biodegradable film for antioxidant preservation at food interfaces. Incorporating pomegranate peel extract into sodium alginate edible coating showed better effects on maintaining the antioxidant properties, inhibiting the internal browning, and decreasing the polyphenol oxidase activity of pear fruit during low-temperature storage [95].

### 3.2. Antibacterial Properties

Fresh fruits and vegetables are one of the main vehicles for foodborne illness caused by pathogenic bacteria, primarily due to their consumption in raw form [96]. Many phenolic compounds demonstrate substantial antibacterial activity, with the primary mechanism involving disrupting the structure and permeability of cell membranes and damaging the bacterial macromolecules synthesis and energy metabolism [97]. Polyphenols interact with the cell membranes of bacteria, disrupt phospholipids or lipid bilayers, affect fluidity, and increase membrane permeability, thereby altering the ion transport systems [98]. Quercetin, catechins, gallic acid esters, and other plant polyphenols have excellent antibacterial properties, due to their inhibitory effects both on Gram-negative and Gram-positive bacteria, attributed to the disintegration of the outer cell membrane and the interrupting of quorum sensing signalling and biofilm formation [99]. Lima et al. [100] evaluated the antibacterial activity of the phenolic-rich pulp and seed extracts of beach-apricot fruit (*Mimusopsis comersonii*) and found that these extracts inhibited all tested microorganisms, particularly *Staphylococcus aureus* and *Salmonella typhimurium*. Makwana et al. [101] investigated the antibacterial effects of six compounds against *Escherichia coli W1485* and *Bacillus subtilis*, which serve as models of common pathogenic microorganisms. Among these compounds, two natural phenolic compounds (resveratrol and cinnamaldehyde) exhibited the most potent antibacterial activity. Previous studies have shown that coatings or films containing phenolic compounds provide better antibacterial properties for preserving fruits. For example, Li et al. [102] found that chitosan films containing tea polyphenol nanoparticles had a stronger ability to inhibit *Escherichia coli* and *Bacillus subtilis*.

### 3.3. Antifungal Properties

Infection by fungal pathogens is considered as the main reason of fruit decay during storage and transport, as fungal pathogens’ growth on raw and processed foods leads to the degradation of sensory properties and nutrient composition, rotting, off-flavors, and odor emissions [103,104]. Studies have shown that phenolic compounds can mitigate the severity of fungal infections due to their chemical structure, concentration, and interactions with substrates. Fruits and vegetables are particularly vulnerable to aggressive fungi, including *Penicillium*, *Botrytis*, *Monilinia*, *Cladosporium*, *Rhizopus*, *Mucor*, and *Alternaria* genera. Phenolic compounds have been studied and proposed as antifungal agents for food preservation, as they can alter biological membrane functionality due to their lipophilic properties and the presence of a hydroxyl group. Phenolics play a role as the uncoupler of oxidative phosphorylation, allowing it to permeate cellular membranes without affecting its integrity [105]. Salicylic acid, a phenolic acid commonly associated with plant growth, has demonstrated antifungal properties against postharvest infections such as *Botrytis cinerea*, *Penicillium expansum*, and *Rhizopus stolonifers*, even at low concentrations. Given its strong antifungal qualities and low toxicity to the environment and human health, salicylic acid is proposed as an alternative to fungicides for treating postharvest infections in apples [104]. Three relevant postharvest fungal pathogens, *Monilinia fructicola*, *Botrytis cinerea*, and *Alternaria alternata* were used to evaluate the antifungal capacity of orange peel polyphenolic extract (OPE). OPE extract at 1.5 g/L inhibited (100%) the mycelial growth and conidial germination of the three target fungi, and the phenolic acids (ferulic and p-coumaric) displayed significantly higher inhibitory capacity [106]. Moreover, flavonoids also inhibit fungal spore germination and have been suggested for controlling fungal pathogens [107].

## 4. Coatings and Films with Phenolic Compounds

Incorporating phenolic compounds into food packaging represents an emerging trend in the packaging industry. The strength of the interaction between polyphenols and polysaccharide molecules can affect the physical properties of the composite film, such as hydrophilicity, extensibility, and microstructure. The main influencing factors include the number of hydrogen bonds formed between plant polyphenols and polysaccharides, the proportion of blending substrate, and the film-forming methods [108,109]. The preparation methods of phenolic compounds combining polysaccharides-based coatings and films would affect the functions and properties of the composite materials. Numerous researchers have studies preparing polysaccharides-based coatings and films containing phenolics by different methods.

### 4.1. The Methods of Adding Phenolic Compounds

#### 4.1.1. Direct Incorporation

The direct incorporation of phenolic compounds into film-forming matrices represents a prevalent approach for preparing polysaccharide-polyphenol composite films or coatings. A substantial body of research employs this methodology to integrate polyphenols into thin films or coatings. Typically, phenolic compounds with poor solubility in water, such as curcumin and ferulic acid, necessitate pre-dissolution in organic solvents, like ethanol solutions, or the addition of surfactants, such as Tween, to facilitate dispersion [27,110,111,112].

#### 4.1.2. Covalent Grafting

Covalent grafting refers to introducing various types of polyphenolic compounds into polysaccharide structures through chemical coupling [113], enzyme-mediated techniques [114], and free radical methods [115]. Research indicates that the coupling efficiency of different polyphenols with polysaccharides varies [116], and the degree of substitution of identical conjugates may exhibit a positive correlation with the barrier, mechanical, and antioxidant properties of the resulting films [117].The performance of polysaccharide films derived from different phenolic acids using identical grafting and film-forming methodologies exhibits variability [118]. The covalent grafting of polyphenols onto polysaccharides can enhance the stability and functional attributes of polyphenols, thereby improving the performance of the resultant films [119]. Compared to simple blending, the covalent cross-linking of polyphenols with polysaccharides to form films results in a significant enhancement of elongation at break and tensile strength [114].

#### 4.1.3. Loading

Due to the limited solubility and stability of most phenolic compounds in water, researchers have employed emulsions and nanoparticle forms to enhance their solubility and stability, as well as to achieve controlled release rates [120,121].

In film-forming matrix systems, the continuous phase is predominantly aqueous; however, numerous phenolic compounds with commendable antimicrobial and antioxidant properties exhibit poor solubility in water. Consequently, many studies have adopted the emulsion approach to disperse phenolic compounds within the film-forming matrix, thereby augmenting their solubility. Nanoscale emulsions, characterized by their diminutive size and augmented surface area, confer superior polyphenol-loading capacity, enhanced solubility, and superior preservation attributes [122]. Employing methodologies such as ionotropic gelation and simple adsorption techniques, phenolic compounds can be fabricated into diminutive nanoparticles. Nanoparticles represent a promising modality for payload, characterized by their commendable stability, solubility, and attributes conducive to controlled release [123,124]. Liposomes constitute another efficacious nanoscale polyphenol carrier, with their highly versatile self-assembled spherical nanostructures, composed of phospholipids, enabling the encapsulation of polyphenols, thereby enhancing their stability and facilitating delayed release [125,126]. Load-bearing systems exhibiting analogous characteristics include microcapsules. These are formed by encapsulating phenolic compounds using diverse wall materials to create minuscule capsules. Incorporating polyphenol microcapsules into films or coatings extends and regulates the release of polyphenols, thereby endowing the films or coatings with enhanced antimicrobial and antioxidant properties [127]. Another distinct encapsulation technique is the use of cyclodextrin inclusion complexes. Cyclodextrins possess a unique barrel-shaped molecular structure with a hydrophobic cavity that can form a stable complex with phenolic compounds, thereby enhancing their solubility and stability in water [128,129]. Metal-Organic Frameworks (MOFs) represent a novel class of crystalline, porous inorganic–organic hybrid materials characterized by their regular porosity and robust chemical properties, rendering them an innovative approach for encapsulating phenolic compounds [130].

Incorporating polyphenols can significantly enhance the performance of films or coatings, with diverse addition methods adequately addressing the fabrication requirements for various polyphenol–polysaccharide films. However, the structural diversity of phenolic compounds may dictate distinct suitable addition methods, an area currently lacking comprehensive investigation.

### 4.2. Preparation of Coatings and Films

#### 4.2.1. Coatings

**Figure 3:**
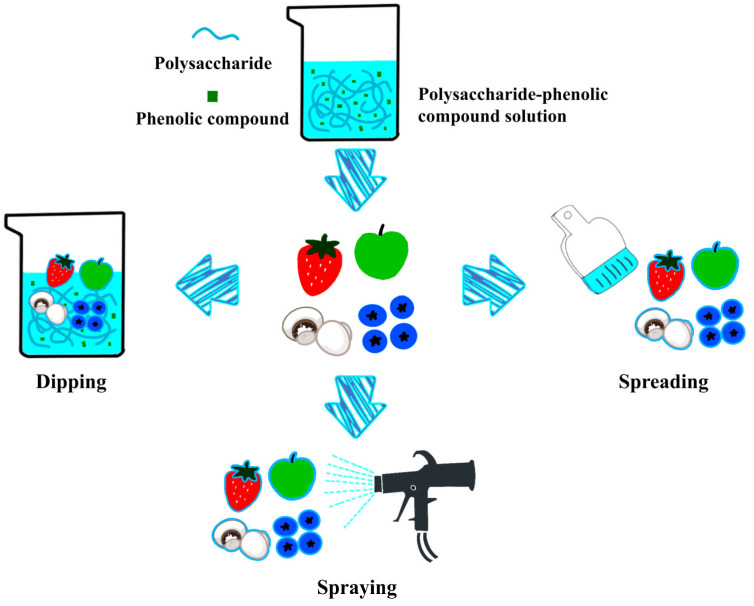
Preparation methods of coatings.

Coating technology is the application of edible materials to the fruit and vegetable surface to form a film with physical barrier properties and physiological inhibition to achieve high-quality preservation. Through the coating process, the coating method is a direct influencing factor for the quality of the coating, which in turn affects the final preservation of the fruits and vegetables. Currently, the commonly used coating methods include dipping, spreading, and spraying (Figure 3).

##### Dipping Method

Dipping is the preferred method for coating the surfaces of irregular fruits and vegetables. This technique is widely accepted due to its ease of operation and cost-effectiveness [131,132]. Applying polysaccharide-phenolic compound active coatings involves three main steps. The first step entails immersing fresh fruits and vegetables in the coating solution (including film-forming polysaccharide and functional polyphenol), with the precise dipping time determined by the specific experiment [133].The subsequent steps include the deposition of excess polysaccharide solution, followed by drainage and the use of hot air to accelerate evaporation and drying of the coating film [134,135]. Certain gel coatings, upon impregnation, can maintain the quality of fruits and vegetables without the necessity of subsequent drying processes [131]. The thickness and morphology of the coating material deposited on the surfaces of fruits and vegetables via the dipping method depend on various factors, including dipping time, extraction rate, dipping-coating cycle, polysaccharide viscosity, and drying conditions. Vacuum impregnation, as opposed to conventional soaking methods, facilitates the formation of a thicker and more efficacious coating on the surface of fruits and vegetables [131,136]. However, dipping may lead to removing the natural wax coating on fruits and vegetables, thereby reducing their original barrier function [137,138].

##### Spreading Method

The spreading method involves applying a high-viscosity polysaccharide–polyphenol solution to the surfaces of the fruits and vegetables using a sterile brush [139,140]. The proposed method is amenable to fruits and vegetables of varying shapes and sizes, offering the advantage of convenient operability [141,142]. Polysaccharide-based solutions are typically performed manually by experienced operators to minimize application errors and variations in ingredient quality, resulting in improved coating uniformity. In addition to the manual application, the coating process can also be executed through mechanical devices equipped with brushes [143]. Furthermore, the type of brush employed exerts a significant influence on the efficacy of the coating. Njombolwana and colleagues [144] conducted a study investigating the impact of using horsehair brushes and synthetic brushes on coating quality and the preservation efficacy of citrus fruits. The experiments revealed that the coatings formed by both brushes exhibited uneven surfaces and many cracks. However, it was observed that coatings produced with horsehair brushes were comparatively smoother, thereby yielding superior preservation outcomes for citrus fruits.

##### Spraying Method

The spraying method involves the dispersion of a coating solution into fine droplets through a nozzle, which is applied to the surface of fruits and vegetables. The coatings formed through this method are comparatively thinner than those produced by the dipping technique and offer the advantage of a more rapid application process, rendering it suitable for commercial production purposes [145]. However, research conducted by Hong and colleagues [146] indicates that the film layers prepared using the spray application method exhibit higher surface roughness and porosity. This phenomenon may be attributed to the aggregation of sprayed droplets. An advancement over traditional spraying techniques is the application of electrostatic force for the depositing of edible coatings. Electrostatic spraying involves the expulsion of negatively charged coating solution under the influence of a high-voltage electrostatic field, which subsequently adheres to the positively charged surface of fruits and vegetables, enhancing uniformity and resulting in the fine particle size of the film-forming material [147]. Furthermore, electrostatic spraying promotes more efficient adhesion to fresh fruit surfaces than conventional spraying owing to the electrostatic interactions between micrometer-sized charged droplets [148].

#### 4.2.2. Films

**Figure 4:**
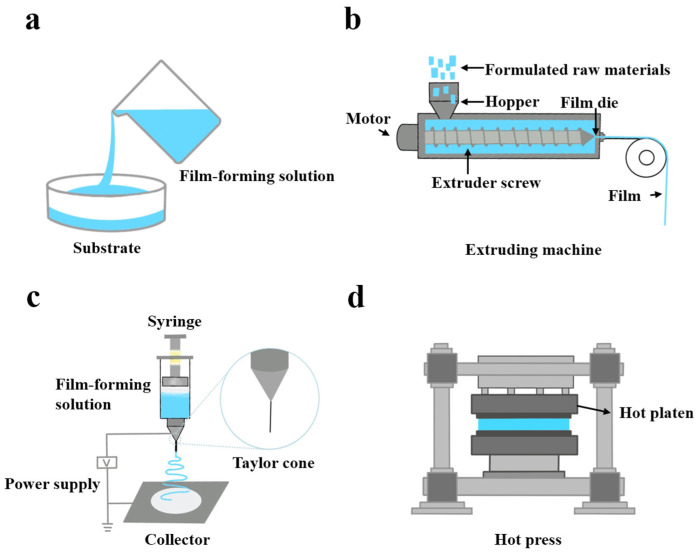
Preparation of polysaccharide-based films. Casting (**a**), Extrusion (**b**), Electrospinning (**c**), Thermo Compression (**d**).

The production of food packaging films mainly involves two methods: the wet process and the dry process. The wet process entails dispersing or dissolving the polymer in a solvent medium before forming the film using casting methods, while the latter consists of hot pressing molding or melt extrusion of powdered polymers (Figure 4). Compared to the traditional wet process, the dry process is more suitable for large-scale continuous industrial preparation of edible films [8].

##### Casting

The casting technique is the most commonly employed method for preparing polysaccharide-phenolic compound films, particularly on laboratory or pilot scales, due to its cost-effectiveness and ease of manipulation. The casting process consists of three main procedures: dissolving, casting, and drying. First, the polysaccharide and phenolic compound are dissolved in a suitable solvent to prepare a film-forming solution. Once the solution is prepared, it is cast into a predefined mold. To achieve a smooth film surface, it is essential to prevent the formation of air bubbles. The final step involves drying, which enhances the molecular interactions among polymer chains and which is crucial for the removal and evaporation of the solvent [149]. The drying time is relative and depends on several factors, including the volume of the film solution, the type of dissolved material, and the drying temperature. During this process, the thickness of the film is regulated based on the amount of solution deposited onto the surface.

##### Extrusion

Extrusion is a prevalent method in the film industry, characterized by its continuous nature and minimal use of solvents at elevated temperatures and under significant shear forces [150]. Films produced via extrusion exhibit distinct physical properties compared to those obtained through continuous casting. The film-forming solution is precisely deposited into a feed hopper and subsequently directed to a heated barrel, which may contain one or two screws. These screws play a pivotal role in homogenizing and compacting the solution, and their design, along with the processing temperature and speed, is meticulously controlled to match the characteristics of the polymer being processed. At the end of the process, the extruded material is compressed, cooled, and molded into the final film [151,152]. In general, due to the high energy input typically associated with the film fabrication process via extrusion, films prepared by this method necessitate using thermally stable components [153,154,155]. However, recent research has indicated that these issues can be ameliorated through optimizing extrusion processes. For instance, the addition of four polyphenols—naringin (NAR), gallic acid (GA), caffeic acid (CA), and quercetin (QUER)—to bio-based high-density polyethylene (bio-HDPE) has successfully enhanced the thermo-oxidative stability of bio-HDPE [156]. Gao et al. [157] successfully fabricated anthocyanin-loaded films by spraying a blueberry extract solution onto starch/polybutylene adipate (PBA) pellets before extrusion blow molding.

As a dry process, extrusion requires a film matrix with low moisture content and it can alter the original structure and characteristics of the film materials [158,159]. This method offers advantages such as reduced processing time, lower energy consumption, and increased efficiency, making it suitable for large-scale commercial production. Furthermore, compared to wet preparation methods, solubility issues are minimized, as solvents are not required.

##### Electrospinning

Electrospinning, a method for producing submicron to nanoscale diameter fibers by ejecting polymer solutions in a high electric field, offers tuneable physical properties such as diameter, density, and alignment, as well as effective encapsulation of active substances, making it a suitable technique for the fabrication of preservative films. Recent studies have explored the application of electrospun fibers in active and intelligent packaging materials [160], as well as in multilayered structures [161], resulting in novel packaging solutions with improved mechanical, barrier, and antimicrobial properties. Furthermore, electrospinning can be combined with layer-by-layer assembly to produce multilayer composite films with stratified structures, which are capable of retarding the release of functional components [162,163]. Additionally, research has discovered that films prepared by the electrospinning method exhibit superior solvent resistance compared to those fabricated by the casting method [164]. This approach offers a promising avenue for developing advanced packaging solutions that can actively respond to environmental changes, extend shelf life, and ensure the safety and quality of packaged products.

##### Thermo Compression

Thermo-compression is a method that involves mixing materials and heating them to a specific temperature, followed by applying pressure using a press machine to form a film [165]. This technique is straightforward and is considered an environmentally friendly and cost-effective fabrication method. Compared to the casting method, films produced by thermo-compression exhibit lower water content and possess reduced water vapor permeability[166,167]. Similar to the extrusion process, thermo-compression requires high temperatures during fabrication, which can lead to partial loss of thermolabile bioactive substances. As observed in Ordoñez’s study [168], the thermolabile ferulic acid experienced a loss of approximately 40% after melt blending and compression molding. Despite the potential loss of active components during the thermo-compression process, technological advancements offer the prospect of mitigating this issue. In Gaviria’s research [169], the addition of laponite was found to ameliorate the loss of anthocyanins caused by thermo-compression. In addition to the concern of active substance loss, studies have indicated that films prepared by thermo-compression may exhibit uneven material distribution. Andretta et al. [170] developed and characterized pH-indicator films based on cassava starch and blueberry residue using thermo-compression, and through optical microscopy, it was observed that the dispersion of blueberry residue within the polymer matrix was irregular. With the growing awareness of environmental protection, thermo-compression is gaining attention for its potential environmental-friendliness and sustainability, leading to an increasing number of studies employing thermo-compression to fabricate preservative films and improve their manufacturing processes.

#### 4.2.3. Layer-by-Layer

Layer-by-layer (LBL) assembly is an effective methodology employed for constructing a diverse array of functional coatings or films, representing a prevalent technique for the fabrication of multilayered films. This approach facilitates the tailored design of the materials and architecture of films. The stability between the layers is achieved through non-covalent interactions, such as electrostatic interactions, hydrogen bonding, coordination bonds, and covalent interactions. Current research on multilayer films for preservation applications predominantly relies on electrostatic interactions. Researchers typically use materials such as chitosan [171], sodium alginate [172], and pectin [173] to create multilayer films or coatings based on electrostatic interactions. The construction of covalently assembled LBL films necessitates a variety of chemical reactions, including the formation of amides or anhydrides through thermal or catalytic processes, amine alkylation reactions, click reactions, aldol reactions, or Schiff base reactions [174]. Compared to blended monolayer films, the LBL assembly technique alters the intermolecular interactions and thermal stability, resulting in multilayered films with enhanced mechanical properties, sustained release characteristics, and antimicrobial efficacy [172,175]. Furthermore, the LBL assembly technique allows for the precise fabrication of coatings with specific barrier properties by modulating the assembly concentration and the number of assembly cycles, which is of considerable significance for applying preservative coatings on a diverse array of fruits [176].

In summary, polyphenols can be immobilized on the surfaces of films or incorporated throughout coatings. Some polyphenols are sensitive to heat, so their bioactive activity may be lost during thermal processing. In this case, non-thermal preparation methods, such as electrospinning or casting methods, should be used to protect their functional properties. The selection of an appropriate preparation method depends on the nature of polyphenols used, the desired properties of the final materials, and the application scenarios of the packaging.

## 5. Improvement of Preservation Properties by Adding Phenolic Compounds

**Figure 5:**
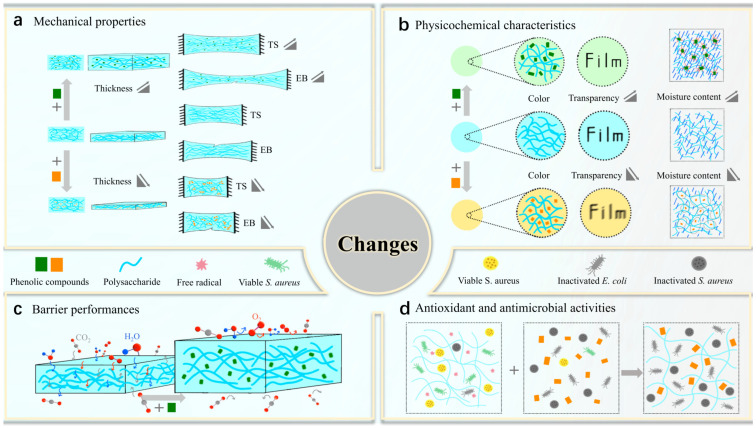
Changes in coating and film properties after the addition of phenolic compounds.

Polysaccharide–phenolic compound coatings and films, as alternative edible materials, must compete effectively with synthetic plastics that are currently in widespread use. The interactions between polysaccharides and phenolic compounds are multifaceted and significantly influence the properties of these coatings and films, such as mechanical properties (tensile strength and elongation at break), physicochemical characteristics (moisture content, solubility, thickness, color, and transparency), and barrier performances (water vapor permeability, oxygen, and carbon dioxide transmission rates), as well as the antioxidant and antimicrobial capacity. The changes in coating and film properties after adding phenolic compounds are illustrated in Figure 5.

### 5.1. Mechanical Properties

The mechanical properties of food packaging coatings and films are of great importance because these packages must remain intact and stable against potential external forces encountered during packaging applications.

#### 5.1.1. Thickness

The thickness of coatings and films impacts their barrier and mechanical properties, as well as tensile strength, which is determined by the total insoluble mass in the film-forming solution and the intermolecular interactions within the films [177]. Generally, the greater the mass of non-soluble substances in the film-forming solution, the thicker the resulting film. The addition of phenolic compounds can increase the thickness of the polysaccharide-based coatings and films. The thickness of the coatings and films increases with the augmentation of polyphenol content, exhibiting a concentration-dependent relationship. For chitosan-pectin films with the addition of tea polyphenols at 2.5% (CPFT1 group), 5% (CPFT2 group), and 15% (CPFT3 group), the measured thicknesses were 0.092 ± 0.001 mm, 0.098 ± 0.005 mm, and 0.109 ± 0.003 mm, respectively [15]. Similarly, as the content of proanthocyanidins increased from 0 to 20%, the thickness of the chitosan-proanthocyanidins (CS-PA) films increased from 0.045 mm to 0.072 mm [178].

However, this pattern may be altered considering the interactions between film components. Research findings by Xue et al. [179] indicate that introducing three phenolic compounds significantly reduced the thickness of the film. Moreover, different types of compounds varied in their extent of altering the thin film thickness, with the films produced using propolis flavonoid-loaded emulsions being the thinnest. This could be attributed to the stronger interaction forces between propolis flavonoids and the film solution or the smaller size of the emulsion droplets formed. Ma et al. [180] demonstrate that the incorporation of potato peel polyphenols did not significantly affect the thickness of the thin film. The disparity in results may be attributed to the varying strengths of interactions between different polyphenols and the film-forming solution, with stronger interactions potentially leading to a more compact film structure.

#### 5.1.2. Tensile Property

Tensile strength is used to measure the strength at the break of coatings and films. Adding phenolic compounds can significantly improve the tensile properties of these films [181]. A substantial body of research indicates that adding an appropriate amount of polyphenols can enhance intermolecular interactions within the film, thereby improving its tensile strength. Bi et al. [178] observed a significant increase in the tensile strength of the film prepared with chitosan (CS) and proanthocyanidins when the proanthocyanidin content was 10% to 20%. Similarly, another study explored the impact of cinnamaldehyde-tannic acid nanoparticles on the tensile strength of CS films. The incorporation of 0.6% polyphenolic nanoparticles resulted in approximately a 5.25-fold increase in the tensile strength of the thin film. This enhancement may be attributed to the strengthening of intermolecular hydrogen bonding between chitosan (CS), cinnamaldehyde (CA), and tannic acid (TA) molecules, as well as the electrostatic interactions between the cationic amino groups (−NH^3+^) of CS and the anionic phenolic hydroxyl groups (Ar-O) of TA [182]. These findings suggest that the influence of polyphenol addition on the tensile strength of films is closely related to the quantity added. An additional intriguing study indicates that the alteration of tensile strength by polyphenols is also contingent upon the incorporation method. Compared to films prepared with rutin in a free suspension, the addition of rutin in the form of liposomes resulted in a significant decrease in tensile strength. The authors attribute this to the distribution of liposomes along the surface and a consequent reduction in intermolecular interactions within the film matrix [125].

#### 5.1.3. Elongation at Break

Elongation at break (EB) is defined as the change in length of coatings and films when stretched to the point of rupture and is used to evaluate the tensile capacity of the film. A higher elongation at break indicates superior flexibility and extensibility. Moreover, EB is indicative of the film’s processability, which is particularly significant in fresh-keeping applications. This property could be effectively improved by phenolic compounds [183]. The variation in EB is dependent on both the concentration of polyphenol incorporation and the type of phenolic compounds. Yerramathi et al. [111] demonstrated that the EB of sodium alginate films progressively decreases with increasing concentrations of ferulic acid. Films with higher concentrations of ferulic acid (35 mg/g and 45 mg/g) exhibited a significant reduction in EB compared to control films. This can be attributed to enhanced crosslinking at certain ferulic acid concentrations. In contrast, under specific concentration conditions, the EB of chitosan films exhibits a gradual increasing trend with the addition of curcumin, which the authors attribute to the formation of hydrogen and covalent bonds, potentially leading to enhanced cohesion [184].

Similar to tensile strength, the method of adding phenolic compounds also influences the change in EB. Films prepared by incorporating rutin in the form of liposomes exhibit a higher EB compared to those with direct addition [125]. However, the results of two additional studies have demonstrated divergent outcomes. With the incorporation of tannic acid, the EB values of chitosan-gelatin films significantly decreased [185]. Upon the incorporation of tannic acid nanoparticles into chitosan films, the EB of the films was also reduced [182]. It is evident that the alteration of the EB in thin films by the addition of polyphenols is influenced by a multitude of factors, including the type of polyphenol, the method of incorporation, the film matrix, and other additives. These factors can all impact the interactions between film components, alter the film’s structure, and consequently affect the EB of the film.

### 5.2. Physicochemical Characteristics

#### 5.2.1. Moisture Content and Water Solubility

Moisture content and water solubility are crucial parameters for food preservation and indicators of coating and film hydrophilicity. Moisture content is a pivotal determinant of a film’s barrier properties, particularly its water vapor transmission rate, which is critical for maintaining equilibrium in the water activity of packaged foods. Furthermore, water solubility significantly affects the migration of membrane components. Excessive solubility can lead to the leaching of coating and film components into food, thereby compromising food safety and palatability.

The incorporation of phenolic compounds substantially influences the moisture content and water solubility of films, contingent on the type and concentration of polyphenols as well as their interactive dynamics with the film substrate. Phenolic compounds may exert an influence on the water content and water solubility of thin films through three potential pathways: (1) hydrophilic or hydrophobic polyphenols fixed within the membrane can interact with water molecules, thereby retaining more or less moisture. Due to curcumin’s hydrophobic nature, a notable decrease in moisture content was observed when the concentration was elevated to 0.24 mg/mL. The water solubility of the films progressively decreased with the addition of curcumin [184]. Conversely, another study involving the incorporation of tea polyphenols into corn starch films revealed an increase in water content compared to films without additive. This enhancement is likely a result of interactions between the adsorptive water in starch and the hydrophilic nature of tea polyphenols, which readily form a complex with water molecules, thereby entrapping a greater amount of water within the starch-tea polyphenol matrix [186]; (2) the phenolic hydroxyl groups carried by polyphenols can interact with the hydrophilic groups of the polysaccharide matrix, thereby limiting the interaction between the film matrix and water molecules; (3) phenolic compounds occupy the voids within the film matrix, thereby reducing the volume available for water. When proanthocyanidins (PA) are added to chitosan (CS) films, the water solubility of the films increases, but their water content decreases. This may be due to the fact that the hydroxyl groups rich in PA can form intermolecular hydrogen bonds with the hydrophilic groups of CS, limiting the intermolecular interactions between CS and water. Furthermore, PA occupies the total void volume of water within the film matrix, further reducing the water content of the CS films [178].

#### 5.2.2. Color and Transparency

The color and transparency of the film directly affect consumer perceptions of packaged food and play a crucial role in food packaging. Due to the inherent color of phenolic compounds, incorporating different phenolic compounds into thin films imparts specific hues to the films, reducing their brightness and causing the films to appear darker [187]. After adding blackberry anthocyanin extract to carboxymethyl cellulose films, the films turned purple and their transparency was reduced [188]. Sun et al. [189] incorporated thinned young apple polyphenols (YAP) into chitosan films and found that as the concentration of YAP increased, the films exhibited a deepening color from a reddish-brown to a yellowish tint and a reduction in transparency, likely attributable to the inherent color of YAP.

Research has found that adding phenolic compounds in different forms can partially mask their inherent colors. Phenolic compounds of different types, due to their distinct functional groups, exhibit variations in light absorption and transmission. Johana’s research indicates that the yellowness index of films with rutin added in the form of liposomes was significantly lower than that of films with rutin added as a free suspension. This is attributed to rutin being entrapped within the liposomes, thereby losing its deep yellow color [125].

The alteration of transparency upon adding phenolic compounds may depend on the type of phenolic compounds. Incorporating proanthocyanidins (PA) notably reduced the light transmittance of chitosan (CS) films, with the ultraviolet-visible light transmittance of CS-PA films gradually decreasing as proanthocyanidin content increased [178]. However, in the report by Yang et al., the addition of tannic acid (TA) and Fe^3+^ enhanced the transparency of pectin films [190].

### 5.3. Barrier Performances

#### 5.3.1. Water Vapor Transmission Rate

The Water Vapor Transmission Rate (WVTR) is a critical metric for assessing the quality of a coating/film, quantifying the rate at which water vapor permeates a specified coating/film area under defined conditions. Proper control of WVTR is essential for regulating the internal humidity of packaged foods, thereby preventing both dryness and excessive moisture absorption. Precise WVTR management, based on the varying moisture sensitivities of different foods, is a key factor in the design of preservation films, ensuring that they create a microenvironment conducive to maintaining food quality.

A substantial body of research has reported on the impact of phenolic compound incorporation on the WVTR of thin films. The effects of incorporating phenolic compounds seem diverse depending on the film matrix and phenolic compounds themselves. Bi et al. [178] found that the WVTR of chitosan film tended to increase with the addition of proanthocyanidins at a level of 5% to 20% of the chitosan. Similar studies have shown that incorporating tannic acid enhances the WVTR of sodium alginate films. This increase in WVTR is likely attributable to the abundance of hydroxyl groups in tannic acid, which can form hydrogen bonds with water molecules, thereby facilitating water vapor permeation [191]. The addition of tannic acid to pectin films, however, results in a decrease in the WVTR, which the authors attribute to the crosslinking between tannic acid and pectin, leading to a denser network structure within the pectin film and consequently reducing the water vapor permeability (WVP) [190]. Apple polyphenols have been found to reduce the WVTR of chitosan films significantly. These effects may be ascribed to the increased thickness and density of the chitosan film induced by YAP, resulting in a more effective barrier against water vapor molecules by either preventing their passage or prolonging the time required for their permeation through the film matrix [189]. The reduction in WVTR was also observed when incorporating guava leaf flavonoids into carboxymethyl chitosan-based coatings [192] and green tea extract (GTE) into rice starch-pectin composite film [193]. Incorporating curcumin in the form of cyclodextrin inclusion complexes into chitosan-cellulose films also significantly reduces their WVTR [129]. Consequently, due to the varying degrees of hydrophilicity of polyphenols and their interactions with film components, the structural changes in the thin films differ, leading to distinct alterations in their water vapor transmission rates.

#### 5.3.2. The Oxygen Transmission Rate

The Oxygen Transmission Rate (OTR) indicates a coating’s or film’s barrier to oxygen penetration. When maintaining the freshness of fruits and vegetables, it is critical to reduce the OTR to limit oxygen flow into the packaging environment. This reduction helps to lower the respiration rate and inhibit enzymatic browning, thus extending the shelf life. Incorporating phenolic compounds can reduce the oxygen permeability of thin films, thereby slowing the oxidation of fruits and vegetables and extending their shelf life [194]. Phenolic compounds can alter the film’s structure and the diffusion pathway of oxygen, and to some extent, can consume oxygen. Singh et al. [195] found that the barrier properties of CS/GA/SC films were significantly enhanced with increasing concentrations of gallic acid (GA) and sodium chloride (SC) in the chitosan (CS) structure, which could be attributed to the reduced tortuosity of the oxygen diffusion path through the film. Similar findings were reported by Xin et al. [196], where the incorporation of curcumin nanoparticles reduced the oxygen permeability of potato starch films.

#### 5.3.3. Carbon Dioxide Transmission Rate

The carbon dioxide transmission rate (COTR) is a critical parameter that measures a coating’s or film’s permeability to carbon dioxide, particularly in food packaging. This characteristic is essential for maintaining the internal gaseous environment of packages containing respiring foods, such as fresh fruits and vegetables, which produce significant carbon dioxide levels. It is imperative for packaging coatings and films to exhibit an appropriate COTR to facilitate the controlled release of accumulated carbon dioxide, thus preventing spoilage and quality loss due to excessive gas buildup. Some studies have indicated that adding phenolic compounds can reduce the COTR of thin films. Xu et al.‘s [197] research demonstrates that films prepared from chitosan (CS)-ferulic acid (FA) conjugates exhibit a significantly reduced COTR compared to chitosan films. Adding thymol and grape seed extract to chitosan films yielded similar results. The authors attribute this to the lower solubility of CO_2_ in the films after the incorporation of thymol and grape seed extract [198].

### 5.4. Antioxidant and Antimicrobial Activities

Incorporating antioxidants into films could enhance disease resistance and antioxidant activity, inhibit browning reactions, and reduce nutrient oxidation [199]. Antioxidant activity, particularly free radical scavenging activity, is a crucial indicator for assessing edible films and coatings. In preparing antioxidant polysaccharide films, the antioxidant capacity is typically directly proportional to the concentration of phenolic compounds within the film. Ulu et al. [183] compared the antioxidant activity of enhanced chitosan (CHS)/β-cyclodextrin (β-CD) biocomposite films containing tannic acid with that of CHS/β-CD biocomposite films devoid of tannic acid. Their findings indicated that increasing the tannic acid content significantly enhanced the DPPH radical scavenging activity of the biocomposite film. Similarly, Bi et al. [178] demonstrated that the DPPH radical scavenging activity of the chitosan-proanthocyanidins film escalated with increasing proanthocyanidins content. The mechanism by which polyphenols enhance the antioxidant performance of composite materials may depend on the delocalization of electrons within the polyphenol benzene ring structure. After interacting with polysaccharides, it induces the ionization of phenolic hydroxyl groups, thereby enhancing the hydrogen supply capacity of the coatings/films system and improving the antioxidant activity of the composite packaging. Furthermore, the antioxidant capacity of polysaccharide-polyphenol composite is due to their ability to scavenge free radicals, nitrogen oxides, hydrogen peroxide, and chelate iron ions [200].

Additionally, incorporating specific phenolic compounds can significantly enhance the antibacterial properties of polysaccharide films. Polyphenols can increase the antibacterial effects through various pathways such as disrupting the stability, permeability, and enzyme inhibition of cell membranes. The antibacterial activity is proportional to the concentration of polyphenols. For instance, Zheng et al. [201] investigated the antibacterial effects of chitosan/starch films with varying concentrations of eugenol against *Escherichia coli* and *Staphylococcus aureus*. They found that the pure chitosan/starch film exhibited no significant antibacterial effect on the two test bacteria; however, the incorporation of eugenol markedly improved the film’s bacteriostatic properties dose-dependently. It is important to note that the type of food spoilage or pathogenic bacteria also influences the antibacterial activity of polysaccharide films. Kaczmarek-Szczepańska et al. [202] reported that adding caffeic acid and ferulic acid to chitosan films did not significantly enhance their inhibitory effect on *Staphylococcus aureus* (Gram-positive bacteria), and the bacterial growth rate remained unchanged. In contrast, incorporating caffeic acid and ferulic acid significantly improved the inhibitory effect on *Escherichia coli* (Gram-negative bacteria), likely due to the differing cell wall structures of Gram-positive and Gram-negative bacteria.

In a multi-component composite material system, the mechanism of antioxidant and antibacterial activity is more complex. For example, when cassava starch, polyphenols, and whey protein isolate are blended, there will be pairwise interactions of the three substances simultaneously. Polyphenols may act as “bridging agents” to enhance the binding affinity of the three substances, or as “blocking agents” to weaken their interactions [203]. Therefore, further in-depth exploration is needed on the research of the polyphenol/polysaccharides/protein ternary composite system, especially the synergistic enhancement or reduction effects of ternary complexes.

## 6. Application of Polysaccharide–Polyphenol Films and Coatings in Fruits and Vegetables Preservation

Fruits and vegetables are susceptible to environmental factors that can lead to spoilage and decay, adversely affecting their marketability and edibility. To address these challenges, there is a growing interest in using polysaccharide-based packaging to replace petroleum-based packaging and to protect fruit and vegetables from quality deterioration caused by both chemical changes and microbial spoilage. Polyphenols –polysaccharide composite packaging combines the advantages of preservative properties both of polyphenols and polysaccharides. Furthermore, the addition of natural polyphenol compounds in packaging materials that could release gradually showed continually protective effects during the postharvest stage. The development of polysaccharide–polyphenol films and coatings has gained numerous attention in recent years, as summarized in Table 1.

### 6.1. Maintaining Sensory and Physi-Chemical Quality and Controlling Decay

After harvest, fruit and vegetables are susceptible to influence by microbial contamination, respiration, oxidative browning, and other factors, which reduce their sensory, physio-chemical qualities, and storage life. The application of polyphenol–polysaccharide-based films and coatings has been shown to effectively preserve the sensory quality of packaged fruits, thereby addressing consumer preferences and enhancing purchasing intent. For instance, Zhang et al. [185] utilized a composite film made from chitosan (CS), gelatin (GL), and tannic acid (TA) for the preservation of fresh-cut apples. This film has demonstrated an effective inhibition of fruit browning; by the end of storage, unwrapped fruits exhibited wrinkled surfaces, in contrast to the relatively plump appearance of the wrapped fruits. Similarly, passion fruits treated with tannic acid-pectin composite coatings displayed a significantly lower shrivel index compared to untreated controls at the end of storage. Untreated fruits presented wrinkled surfaces, while those subjected to the coating process maintained a relatively turgid appearance [190]. Additionally, Halim et al. [206] developed quaternary composite films by incorporating tannic acid into chitosan, gelatin, methylcellulose, respectively, and applying them to the preservation of cherry tomatoes and grapes. The study monitored the appearance and freshness of the fruits over a 14-day period, revealing that treatment with the composite film significantly reduced weight loss and the browning index, thereby enhancing the freshness of both cherry tomatoes and grapes. Notably, the tannic acid-treated biopolymer films showed more promising effects than the untreated films. The adding of phenolic compounds effectively modifies the WVTR and gas transmission rate (O_2_, CO_2_) of composite films, which improves specific water-blocking properties, effectively weakens the transpiration and respiratory rate in fresh food during storage, and keeps the moisture and freshness of fruits and vegetables.

Incorporating phenolic compounds with polysaccharides-based coatings or films possesses effective antimicrobial effects. In both cases, phenolic substances may migrate to food or headspace in a gradual diffusion manner or contact with food and microorganisms directly, thereby exerting a prevention of spoilage microorganisms. Jiao et al. [115] grafted chlorogenic acid (CGA) to chitosan (CS) using a free radical mediated method, and the weight loss and decay rate of the peach fruits were decreased significantly by coating with the CS-CGA conjugate. The strawberries coated with pectin-grape pomace extract showed higher firmness and titratable acidity, lower weight loss and microbial counts [211]. Fresh-cut apples coated with CMCS-GLF (carboxymethyl chitosan-guava leaf flavonoids) could limit the growth of bacteria, and the addition of high-dose GLF caused a more positive effect [192]. Treatment with pectin-based coatings and lemon byproduct extract significantly reduced the value of the total bacterial count (2.58 log CFU g^−1^) and preserved the quality of fresh-cut carrots [210]. Chitosan–catechin coating treatment significantly reduced rot caused by *Penicillium citrinum* and *Aspergillus niger* and maintained the freshness of satsuma oranges [112]. 

### 6.2. Improving Nutritional Quality

Besides extending shelf life and improving physiochemical quality, polyphenol–polysaccharide composite packaging presents a promising solution for modern consumers who prioritize nutritional value in fresh fruit and vegetables. Polyphenol–polysaccharide films have demonstrated notable efficacy in mitigating the loss of nutritional components in packaged fruits. Through their antioxidant properties, these films play a crucial role in preserving the nutritional integrity during storage by delaying the degradation of nutrients and bioactive compounds. Furthermore, polyphenols also provide a protection against any potential oxidative stress points present after postharvest and endow the composite films/coatings with certain free radical scavenging capacity, thereby enhancing their nutritional value and antioxidant activity. The study conducted by Wang et al. [192] shows that fresh-cut apples treated with a carboxymethyl chitosan coating enriched with guava flavonoids (CMCS-GLF) exhibit significantly higher total phenolic and ascorbic acid content over a 12-day storage period compared to the untreated control group and pure CMCS coating samples. Riaz et al. [204] investigated the impact of a chitosan (CS)-based apple pomace polyphenol (APP) composite coating on the storage quality of strawberries. During the storage period, the CS-APP composite coating effectively mitigated the decline in total phenolic content, flavonoid content, and total soluble solids, which was superior to the pure CS coating. In the study conducted by Liu et al. [113], a gallic acid-grafted chitosan film (GA-g-CS) showed significant efficacy in preserving the postharvest quality of white button mushrooms. This film effectively reduced the respiration rates and browning in the mushrooms postharvest, while concurrently enhancing their total phenolic content and the activity of antioxidant enzymes. Chen et al. [219] incorporated tea polyphenols (TP) into a composite film-forming solution of xanthan gum (XG) and hydroxypropyl methylcellulose (HPMC) to fabricate an XG-HPMC-TP complex film (XHT) for the preservation of fresh-cut green bell peppers. After 8 days of storage, the retention of vitamin C in samples packaged with XHT was 127.81% higher compared to those that were not packaged.

### 6.3. Develping Intelligent Packaging

In the context of a globalized food supply chain, there is a growing consumer demand for freshness and safety in food products. To meet these expectations, intelligent packaging technology has been developed to monitor food storage conditions in real-time, ensuring that products maintain optimal quality throughout the supply chain. Polyphenol–polysaccharides incorporation has opened up new avenues for intelligent packaging development. Active and intelligent dual-functional food packaging could be prepared based on phenolic compounds and polysaccharides, realizing the monitoring and controlling of fresh food quality [221]. The usage of some phenolic pigments (anthocyanins, flavonoids, and curcumin, etc.) as sensors or tags has shown excellent advantages, including high safety, pH-sensitivity, easy production, and application. Anthocyanins, a category of phenolic compounds that are sensitive to structural changes in response to environmental pH variations, enable the film to alter its color according to the pH level it encounters. This color-changing property can be harnessed to visually assess the spoilage of fruit products. Sganzerl et al. [188] developed a bioactive and pH-sensitive film based on carboxymethyl cellulose and blackberry-rich anthocyanin extract. Zhao et al. [208] developed a multifunctional chitosan/tannic acid/corn starch (CHT/TA/CS) bilayer smart film that exhibited reversible and pH-responsive bending deformation. In preservation experiments, this film extended the storage time of bananas from 3 to 6 days and reduced weight loss by 14% compared to the untreated group. Other environmental parameters, such as temperature and gas along the supply chain, also could be monitored by phenolic compounds. Thermochromic beads prepared by combining flavonoids (extracted from red cabbage), alginate, fatty acid, and lecithin for smart packaging showed a very good result in monitoring the freshness of fresh-cut salad and Brussels sprouts [222]. Four different types of gas sensing films were prepared using anthocyanin, curcumin, soluble starch, polyvinyl alcohol, and polyethylene glycol bis (3-aminopropyl) ether. The addition of natural pigments increases the water content and solubility of the films and this gas-sensitive films successfully classified strawberry freshness into fresh, sub fresh, and spoiled [223]. These findings provided novel insights into the development of polyphenol–polysaccharide-based dual-functional intelligent packaging materials.

## 7. Conclusions and Future Prospects

Polyphenol–polysaccharide composite food packaging represents an emerging technology for preserving the freshness of fruits and vegetables, demonstrating significant preservative effects and developmental potential. This technology effectively improves films/coatings’ mechanical and physiochemical properties and strengths their antioxidant and antimicrobial characteristics. Studies have indicated that polyphenol–polysaccharide composite films can reduce weight loss, maintain nutritional content, inhibit microbial growth, and decelerate the senescence of fruits and vegetables. Notably, combining phenolic compounds with a variety of polysaccharides can monitor the freshness and quality changes during storage, making them a safe, intelligent, and environmentally-friendly choice for fruit and vegetables packaging. However, the future of polyphenol–polysaccharides films/coatings still present several challenges.

1. Analyzing the mechanism of polyphenol composite packaging in preserving fruit and vegetables with high-throughput omics technologies is a challenge. Based on the combined analysis of transcriptomics and metabolomics, it is beneficial to reveal the improving preservation mechanism of polyphenol–polysaccharides composite film relating to the physiological metabolism, disease defense, and regulatory pathways of fruit and vegetables.
2. Polysaccharides have been efficiently employed to develop binary and ternary blends with other substances. The optimal blending proportions are crucial to develop very good packaging materials. Moreover, combining composite films/coatings containing polyphenols with other physical-field storage methods to explore compounding preservation techniques and achieve complementary preservative advantages is also a research direction.
3. Regarding the direct contact between coatings/films and food, the convenience of removing coatings/films from the surface of fruit and vegetables also needs to be examined. Most notably, as the concerns surrounding the safety and migration characteristics of materials have emerged, there are raising potential health risk issues necessitating further research to evaluate their safety. Simulating the migration of phenolic compounds under different conditions is necessary to evaluate whether it possesses negative influences.
4. Nowadays, research is mainly focused on the preservative effects of the composite packaging on fruit and vegetables under a static storage environment. Its application along the dynamic supply chain of fruit and vegetables, where environmental factors under dynamic changes can have a significant difference, needs to be considered.

## Figures and Tables

**Table 1:** The application of polysaccharide coatings and films containing phenolic compounds in fruits and vegetables preservation.

Polysaccharide Matrix	Phenolic Compounds	Application	Preservation Effect	References
Chitosan	Chlorogenic acid	Peaches	Inhibiting decay index and respiration rate, maintaining firmness and ascorbic acid content	Jiao et al. [115]
Chitosan	Apple peel polyphenols	Strawberries	Decreasing decay rate, delaying softening, limiting weight loss, maintaining higher ascorbic acid content, and antioxidant capacity	Riaz et al. [204]
Chitosan	Gallic acid	White button mushrooms	Reducing the respiration rate and the degree of browning, increasing the antioxidant status	Liu et al. [113]
Chitosan	Tannic acid	Bananas, grapes	Extending the shelf life through its excellent water vapor and oxygen barrier	Liu et al. [205]
Chitosan	Catechin	Satsuma oranges	Reducing rots caused by *Penicillium citrinum* and *Aspergillus niger*; prolonging the preservation time	Cheng et al. [112]
Chitosan/gelatin/methylcellulose	Tannic acid	Cherry tomatoes, grapes	Alleviating weight loss and browning	Halim et al. [206]
Chitosan/gelatin	Tannic acid	Fresh-cut apples	Decreasing weight loss and malondialdehyde content, delaying browning degree	Zhang et al. [185]
Chitosan/gum Arabic	Cleistocalyx operculatus extract	Mangoes	Extending the shelf life by limiting the availability of oxygen and impeding the diffusion of CO_2_	Dac et al. [207]
Carbonxymethyl chitosan	Guava leaf flavonoids	Frush-cut apples	Alleviating weight loss and browning, keeping firmness, limiting bacterial growth	Wang et al. [192]
Starch/chitosan	Tannic acid	Bananas	Prolonging the storage time from 3 to 6 days, reducing weight loss by 14%	Zhao et al. [208]
Pectin	Tannic acid	Passion fruits	Extending the shelf life, limiting weight loss and wrinkling	Yang et al. [190]
Pectin	Karonda polyphenols	Ber (*Zizyphus mauritiana* Lamk.)	Maintaining the quality and nutritional potential by reducing spoilage, delaying ripening, and keeping higher antioxidant activity	Kaur et al. [209]
Pectin	Lemon byproduct extract	Fresh-cut carrots	Preserving the physiological parameters and valuable compounds, reducing O_2_ consumption and physiological activity, delaying senescence, improving microbiological stability	Imeneo et al. [210]
Pectin	Grape pomace extract	Strawberries	Preserving the antioxidant capacity, delaying the degradation of bioactive compounds, inhibiting the growth of yeast and mold	Kaynarca et al. [211]
Pectin	Pomelo flavedo extract	Jackfruit bulbs	Alleviating the changes in weight loss and color parameters, inhibiting the spoilage	Nhi et al. [212]
Pectin	Jackfruit extract	Tomatoes	Improving the shelf life by reducing the severity of fungal infection and alleviating the changes in firmness and color attributes	Aguilar-Veloz et al. [213]
Pectin/chitosan	Procyanidins	Grapes	Slowing down the water loss, suppressing the browning	Tie et al. [214]
Pectin/chitosan	Epigallocatechin gallate	Strawberries	Preventing the spoilage and weight loss during prolonged storage	Fu et al. [215]
Pectin/CMCS	Blueberry anthocyanins	Strawberries	Delaying the spoilage of post-harvest fruits and prolonging the shelf life	Sun et al. [216]
Carboxymethyl cellulose	Tannic acid	Strawberries, mangoes, cherries	Prolonging the shelf life of fruits with lower mass loss rate and decay rate, and higher hardness and SSC	Zhao et al. [217]
Carboxymethyl cellulose	Blackberry anthocyanins	Cherry tomatoes	Maintaining firmness, alleviating weight loss	Sganzerla et al. [188]
Carboxymethyl cellulose	Tannic acid/ginkgo biloba leaf extract	Strawberries, cherries and mangoes	Reducing the decay rate, delaying softening	Yang et al. [218]
Cellulose/chitosan	Curcumin	Banana, tomato and apple slices	Extending shelf life of fruit slices by reducing microbial invasion, water loss and oxidation	Zhou et al. [129]
Xanthan gum/ Hydroxypropyl methylcellulose	Tea polyphenols	Fresh-cut green bell peppers	Delaying the ripening of bell peppers, maintaining the vitamin C content	Chen et al. [219]
Konjac glucomannan	Tannic acid	Bananas	Inhibiting the respiratory metabolism, delaying softening	Deng et al. [220]
Sodium alginate	Pomegranate peel extract	Pears	Maintaining higher antioxidant properties, suppressing internal browning	Megha et al. [95]

## Data Availability

No new data were created or analyzed in this study. Data sharing is not applicable to this article.
